# *Mythimna separata* herbivory primes *Coix* resistance in systemic leaves

**DOI:** 10.1371/journal.pone.0313015

**Published:** 2024-11-04

**Authors:** Bo Fan, Yongkuan Liu, Rongkun Wen, Lanfen Kong, Xue Wang, Jingxiong Zhang, Jing Li, Yan Qin

**Affiliations:** 1 School of Food and Pharmacy, Yunnan Light and Textile Industry Vocational College, Kunming, China; 2 School of Biology and Chemistry, Minzu Normal University of Xingyi, Xingyi, China; 3 Department of Economic Plants and Biotechnology, Yunnan Key Laboratory for Wild Plant Resources, Kunming Institute of Botany, Chinese Academy of Sciences, Kunming, China; Hainan University, CHINA

## Abstract

*Coix lacryma-jobi* L. belongs to family Poaceae, is widely cultivated in tropical Asian countries for its nutritional and medicinal values. *Coix* is often threatened by lepidopteran such as *Mythimna separata* during its life cycle, resulting in severe yield reduction. Insect feeding can trigger defense signaling and increased defense responses in many other crops, yet little is known about whether simulated armyworm feeding on *Coix* leaves could induce anti-herbivory responses and whether armyworm feeding could activate priming in systemic leaves. In this study, *Mythimna separata* simulated herbivory elicited increased jasmonic acid (JA) level, JA-Ile (JA-isoleucine conjugate) and altered transcriptome in the *Coix* leaves. Meanwhile, *M*. *separata* simulated herbivory in local leaves primed the systemic leaves for increased accumulation of jasmonic acid and enhanced resistance to *M*. *separata*. Consistently, transcriptome analysis showed the systemic leaves were primed, which were up- or down-regulated comparing with the non-primed systemic leaves. In this study, we first reported *Mythimna separata* simulated herbivory induced increased defense response in leaves of *Coix*, *also Mythimna separata* herbivory primed *Coix* resistance in systemic leaves. This study provides new insight into the regulation of defense responses of *Coix* against *M*. *separata* and the ecological function of priming in *Coix*.

## Introduction

Plants have evolved a variety of defensive strategies against herbivores over a long period of time [1]. Plant defenses are often classified into constitutive defenses and inducible defenses. With inducible defenses, plants managed their resources more flexibly between defense and growth in most cases, activating the anti-herbivore defense only when necessary.

To achieve an efficient and timely response, plants evolved sophisticated systemic signaling pathways to communicate from local tissue to the systemic tissue in response to wounding or herbivory within minutes [2,3]. Many studies have documented the local and long distance defense responses to wounding and herbivory in maize [4–6]. Simulated *M*. *separata* herbivory induced defense-related responses such as jasmonic acid (JA) accumulation and transcriptomic rearrangement in systemic leaves in maize [7].

Priming of defense is an adaptive strategy that enhances the readiness of induced defense. When plants are trigged by warning signals such as herbivore attack from arthropod, stimuli from pathogens, and other abiotic cues in the future, plants are allowed to induce faster and stronger defense response and result in increased resistance or stress tolerance [8]. Priming can be maintained throughout the life cycle of a plant and across generations, thus representing a type of plant immunity memory [9]. The priming of defense against biotic stresses have been well studied in many plant. After the first report of priming between plant and insect in maize [10], significant advances have been made in the defense priming to plant-insect interaction [11]. Meanwhile, the underlying molecular mechanism of priming are gradually being elucidated, such as mitogen-activated protein kinase (MAPKs) [12], epigenetic modification of chromatin [13,14].

*Coix lacryma-jobi* L., commonly known as Job’s tear, adlay or Chinese pearl barley, as an annual or perennial crop is widely cultivated in Asia countries. The seeds of *Coix* are popular nutritional supplemental material and have exhibited many properties in traditional medicine [15–20]. *Coix* often suffers from the chewing insects such as *Mythimna separata*, which can eat most of the leaves resulting in the yield reduction. It is well known that phytohormone JA plays a vital role in regulating the defense of plants [21–24]. The impairment of the JA or JA-Ile (JA-isoleucine conjugate) biosynthesis genes in tomato, Arabidopsis and tobacco can increase their susceptibility to insects [25–27]. Many research work had been carried out in maize, for example, mechanical wounding can rapidly induced the increase of JA and applying the oral secretion (OS) of *M*. *separata* to fresh mechanical wounding (to simulate the insect feeding) induced higher JA [28]. Besides the phytohormone regulation, transcriptomic rearrangement is also important for the defenses of plant. Qi et al., found that simulated *M*. *separata* feeding on maize induced distinct transcriptional response [28]. Also, RNA-Seq analysis exhibited that maize responds to Asian corn borer *Ostrinia furnacalis* feeding with transcriptional regulation of numerous genes, including JA signaling and metabolites [29,30].

So far, little is known about whether the *M*. *separata* feeding on *Coix* leaves induces the defense responses in the systemic leaves and whether the *Coix* systemic leaves could be primed for enhanced resistance to *M*. *separata* attack. In this study, we first show that simulated *M*. *separata* feeding can induce increased JA/JA-Ile and altered transcriptome in systemic leaves in *Coix*. The herbivory feeding assays indicate that *M*. *separata* feeding on *Coix* seedlings primed the systemic leaves for enhanced *M*. *separata* resistance. Moreover, the response of the primed systemic leaves was further demonstrated via transcriptomic changes. Maintenance of primed defensive state in the crop field could be a promising strategy for integrated pest management with enhanced defense activation and efficient resource management. This is the first study showing that the simulated herbivory of *Coix* primed systemic defense responses. This study sheds light on the temporal and spatial regulation of induced resistance of *Coix* against lepidopteran herbivory and provides new insight into the ecological function of priming in *Coix*.

## Materials and methods

### Plant growth and oral secretion collection

The seeds of *Coix* (landrace XRBK) were purchased from a local seeds store in Lutun town, Xingyi city. The seeds were germinated in 12-cm-diameter plastic pots filled with commercial potting soil and vermiculite (about 7:1 ratio) under natural light conditions (about 12–14 h day length) in a greenhouse (25±4°C day, 20±4°C night). Approximately 30-day-old plants, when the third leaves were fully expanded from the whorl, were used for pretreatment. KeYun Pests (https://shop101732681.taobao.com) provided the eggs of *Mythimna separata*. *M*. *separata* larvae were reared on *Coix* until the third to fifth instar for the collection of OS, about 100uL of OS was collected from one larva at a time. *M*. *separata* larvae reared on maize for first instar were used for the priming experiment. Storkbill forceps were used to gently squeeze the caterpillars to provoke regurgitation, and OS were collected on ice with a pipette and immediately centrifuged to obtain supernatant, which was divided into small aliquots before being stored at −80°C.

### Plant treatments, sample collection and herbivore bioassays

To examine whether simulated herbivory induces JA responses in the local *Coix* leaves, the JA and JA-Ile were determined. The fourth leaves of plants were non-pretreated or pretreated with 20uL of *M*. *separata* OS (OS was applied to rows of wounds generated by a pattern wheel, four rows of wounds generated by a pattern wheel). Leaf samples were harvested at 1h and 6h on the whole fourth leaves 4, immediately, frozen in liquid nitrogen. All samples have six replicates.

To study the transcriptomic response to simulated *M*. *separata* herbivory in *Coix* local leaves. The third leaves of *Coix* were untreated, the fourth leaves were treated with W+OS. Plants without any treatment were used as controls. The fourth leaves were collected after 1h and 6h, immediately frozen in liquid nitrogen, and stored at -80°C for RNA-seq. Three replicates were conducted for each sample.

To examine whether *M*. *separata* herbivory primes systemic leaves for increased JA and JA-Ile. *Coix* third leaves were infested with M. separata larvae (one/plant) for four days and then remove the caterpillars. After resting for 4d, the fourth leaves were treated with W+OS, samples of fourth leaves were collected at 0h, 1h and 6h for analyzing JA and JA-Ile contents. While in the control group, the third leaves were untreated, the fourth leaves were treated with W+OS and were collected at 0h, 1h and 6h for analyzing JA and JA-Ile contents.

To examine the effect of priming on *Coix* resistance to insect herbivory, *Coix* third leaves were pretreated by wounding and OS (W+OS) 20 μL of *M*. *separata* once a day for 3days; in the control group, no pretreatment was done. After 4d or 8d, for each *Coix* plant of both control and pretreatment groups, *M*. *separata* larvae (one neonates/plant, first instar, which were reared on maize leaves since hatch) were enclosed in a clip cage fixed on the fourth leaves and allowed to feed for 3d before insect masses were recorded. Each group contained 20 replicate *Coix* plants. This experiment was repeated three times.

To study the effects of simulated *M*. *separata* herbivory-induced priming, the third leaves of *Coix* plants were pretreated with 20 μL of *M*. *separata* OS at rows of puncture wounds generated by rolling a fabric pattern wheel (W+OS pretreatment), and these treatments were repeated once a day for another 2 days. Plants without any pretreatments were used as controls. After resting for 4d, both control and pretreated plants were treated with W+OS on the fourth leaves by immediately applying 20 μL of *M*. *separata* OS to rows of wounds generated by a pattern wheel. Leaf samples were collected 1h and 6h post-treatment on leaf 4, immediately frozen in liquid nitrogen, and stored at −80°C for RNA-seq. Three replicates were conducted for each sample.

### Phytohormone profiling

JA and jasmonic acid–isoleucine conjugate (JA-Ile) were quantified according to the method described previously [31]. In short, 150 mg of frozen leaf powder was extracted with 1ml ice-cold ethyl acetate spiked with 20 ng D_6_-JA and 5 ng ^13^C_6_-JA-Ile. After centrifugation at 13,000 g for 10 min at 4°C, about 900 μL of supernatants were transferred to fresh 2-ml microfuge tubes. Each pellet was re-extracted with 0.5 ml of ethyl acetate and centrifuged, and the supernatants from each sample were combined. The supernatants were evaporated to dryness on a vacuum concentrator (Eppendorf). The residues were re-suspended in 0.5 ml of 70% methanol (v/v) and centrifuged to clarify phases. Following centrifugation, 100 μL of supernatants were pipetted into glass vials and then analyzed by HPLC-MS/MS (LCMS-8040 system, Shimadzu).

### RNA extraction and data analysis

Total RNA was extracted from ground leaf samples using TRIzol reagent (Thermo Fisher Scientific), and the RNA quality, purity, and concentrations were determined using a spectrophotometer (Nano-Drop 2000c, Thermo Fisher Scientific). Sequencing was performed at 5 G depth on a HiSeq2500-PE125 platform (Illumina) and the resulting sequences were trimmed based on quality scores and mapped to *Coix* reference genome sequence [32]. We used HISAT2 [33] to map the transcripts and DESeq2 [34] to identify DEGs. The expression levels of the genes from the *Coix* transcriptomes were calculated and normalized to TPM (transcript per million) values. The genes with a false discovery rate (FDR) less than 0.05 and an absolute value of log_2_^(TPM of treatment/TPM of control)^ greater than 1 were selected as DEGs for further analysis [35].

### Validation of RNA-seq by qPCR

Five genes, each with three biological replicates, were selected for the validation of RNA-Seq by qRT-PCR. Total RNA samples (0.5ug) were reversed-transcribed using oligo (dT) and Superscript II reverse transcriptase (Thermo Fisher Scientific) in a total volume of 10uL. The program was as follows: 42˚C for 60 min and 70˚C for 15min. The cDNA samples were then diluted to 25uL and kept at -20˚C. qRT-PCR was performed using a CFX Connect real-time system (Bio-Rad) using the Talent qPCR Premix (SYBR Green) kit (TIANGEN Biotech, Beijing, China) following the manufacturer’s instructions. The specific primers were designed online (https://www.ncbi.nlm.nih.gov/tools/primer-blast/) according to the following parameters: primer melting temperature of 57–63˚C with the optimized 60˚C, the PCR product size of 90-180bps. The specific primer sequences of the selected genes for qRT-PCR validation were designed based on the divergent regions among the genes and listed in S1 Table. Melting curves were assessed and only primers with single peak were selected. Three technical replicates were used for the samples with 18S rRNA as the reference gene and the quantification of qPCR results for each unigene were calculated using the delta-delta Ct (2^-ΔΔCt^) method. For each gene, a linear standard curve was constructed using a serial dilution of a specific cDNA standard. The transcript levels of all unknown samples were determined according to the linear standard curve. PCR amplication efficiency (E) was calculated as follows: E = (10^[-1/slope]-1^)×100. Each PCR reaction volumes were set at 20uL. The mix contained 10uL of 2×Talent qPCR PreMix, 0.6uL of forward and reverse primers (10uM), 8.7uL of RNase-free ddH2O and 0.7uL of cDNA template. PCR cycling was performed according to the following programs: 3min at 95˚C followed by 40 cycles of 5s at 95˚C and 15s at 60˚C. Melting curve cycling consisted of: 65˚C for 5s and then 0.5˚C increment for 5s until 95˚C [36].

### Statistical analysis

Data of herbivore bioassay were analyzed with Student’s t-test. Analyses on the contents of phytohormones were performed using oneway analysis of variance (ANOVA) and significance was determined by post hoc test (P<0.05). Gene ontology enrichment was performed with the topGO (https://bioconductor.org/packages/release/bioc/html/topGO.html). The enrichment results and Venn diagrams were plotted using web-based tools (http://www.bioinformatics.com.cn and http://bioinfogp.cnb.csic.es/tools/venny/).

## Results and discussion

### Simulated *M*. *separata* herbivory-induced defense responses in local *Coix* leaves

**Fig 1:**
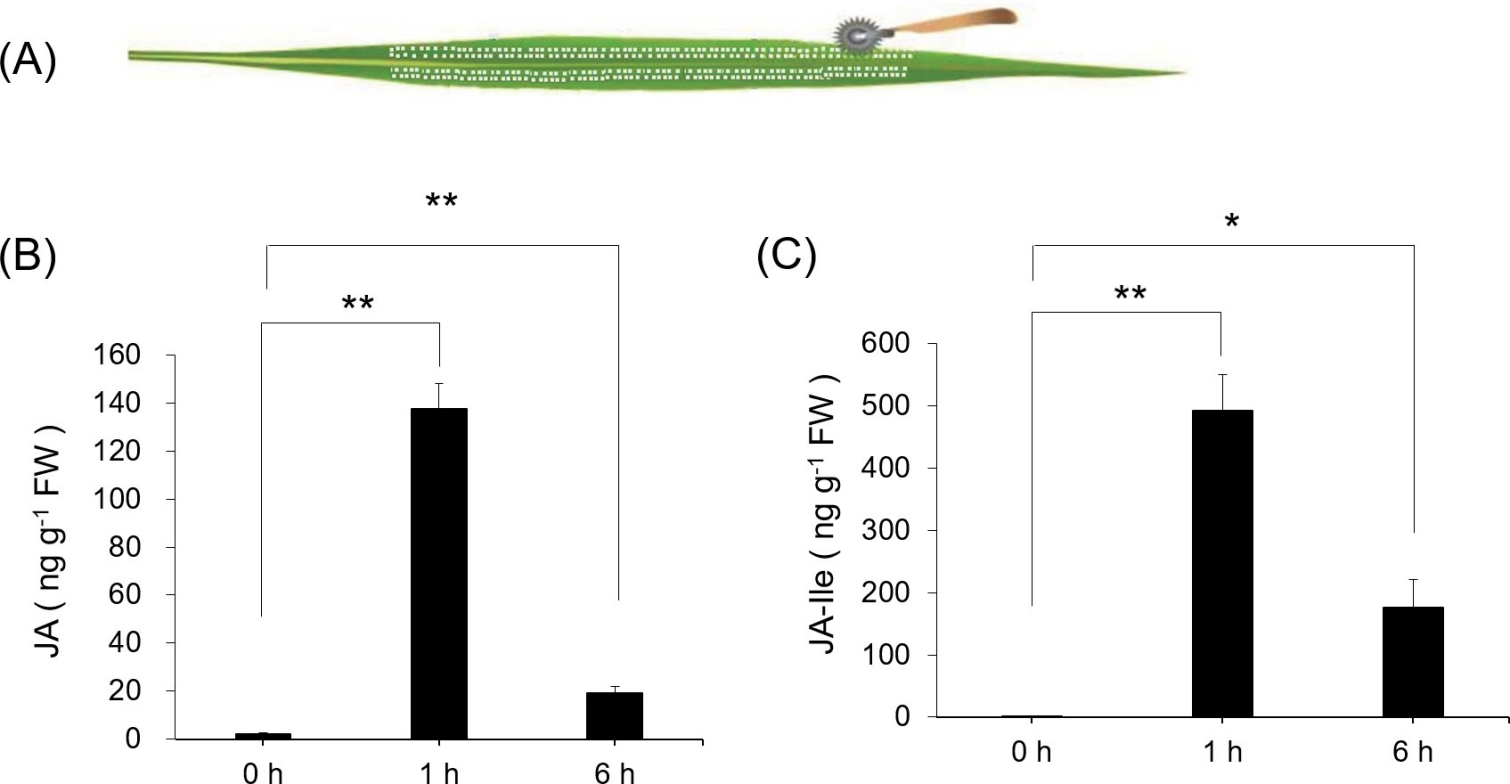
Simulated herbivory of *Coix* seedlings with *M*. *separata* OS by pattern wheel and the levels of JA and JA-Ile. The leaves of *Coix* seedlings were treated with 20μL of *M*. *separata* OS to the wounds generated by a pattern wheel (one row a day) for three consecutive days (W+OS treatment, pretreatment group) (A). JA (B) and JA-Ile (C) levels were measured after W+OS treatment. The third leaves were pretreated with OS, the third leaves were collected at 0h, 1h and 6h, the leaves harvested at 0h were control groups. Asterisks represent significant differences between treated and control group (Student’s t-test; *, p<0.05, **, p<0.01). Data are mean ±s.e. (n = 6). FW, fresh weight.

Simulated herbivory were conducted on *Coix* to investigate the JA levels of the leaves. Since *M*. *separata* herbivory is not easy to control, simulated herbivory was performed by rolling a pattern wheel and immediately applying 20μL of *M*. *separata* OS to the wounds (W+OS, illustrated in Fig 1A) for three consecutive days on the third leaves, then the third leaves were collected at 0h, 1h and 6h, the leaves harvested at 0h were control groups. It was found that the levels of the JA accumulation were 140- and 20-fold at 1h and 6h compared with the control group, respectively (Fig 1B). Similarly, JA-Ile showed about 500- and 200-fold induction at 1h and 6h compared with the control group (Fig 1C).

### Transcriptomic response to simulated *M*. *separata* herbivory in *Coix* local leaves

**Fig 2:**
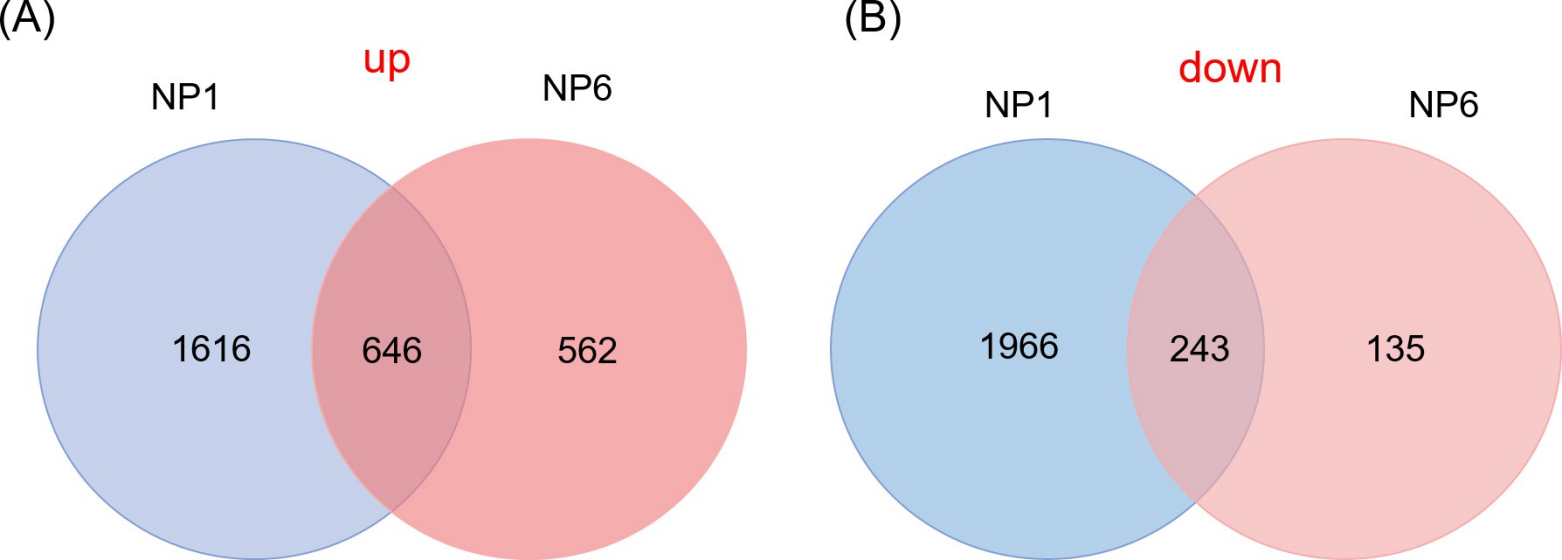
*Coix* leaves exhibit large response to simulated herbivory. *Coix* leaves (fourth leaves) were treated with OS, and the fourth leaves were collected at 1h (non-primed, named NP1) and 6h (non-primed, named NP6). The fourth leaves of untreated *Coix* leaves were harvested as controls. Venn diagram depict the numbers of (A) upregulated and (B) downregulated DEGs of *Coix*. Details of the DEGs of the Venn diagram can be found in the S2 Table.

To study the transcriptomic response to simulated *M*. *separata* herbivory in *Coix* local leaves. The third leaves of *Coix* were untreated, the fourth leaves were treated with W+OS. Plants without any treatment were used as controls. The fourth leaves were collected after 1h and 6h and analyzed with RNA-seq. Totally, 6,057 differentially expressed genes (DEGs) were obtained in this transcriptome sequencing. About 4,471 DEGs were detected after 1h OS treatment and among them, 2,262 genes were up-regulated and 2,209 were down-regulated. Whereas 6h after OS treatment, the leaves induced less transcriptional changes, only 1,586 DEGs were detected, among them 1,208 genes were up-regulated and 378 were down-regulated (S2 Table). Among them, there are 646 genes commonly upregulated and there are 243 genes were downregulated at 1h and 6h after OS treatment (Fig 2A and 2B). We further analyzed top 30 most upregulated genes at 1h and 6h after OS treatment, 3 genes were commonly regulated by simulated herbivory, of which only one (rhomboid family protein) was annotated, indicating the *Coix* regulated various unique genes in response to simulated *M*. *separata* herbivory. Of the 30 most downregulated genes, 8 genes were commonly regulated by simulated herbivory, including serine/threonine phosphatase 2C and wounding repair regulator- Flightless I [37]. The quantitative expression of the DEGs in control, NP1 and NP6 groups were compared.

As an annual or perennial crop, *Coix* is widely cultivated for its nutritional and medicinal values. Previous studies appeared mainly concerning the genetic diversity of *Coix* [38–40]. The first *De novo* transcriptome study of *Coix* identified *Coix* tissues-specific genes and analyzed the prolamin and vitamin E biosynthesis-associated genes [41]. The study of Miao first characterized the response of *Coix* to drought stress [36]. Although the transcriptome work of Miao has been made concerning uncovering abiotic stress such as drought in *Coix*, how this species responds to the biotic stress such as herbivory has not been studied in any detail at the transcriptome level. In this study, we first investigated the simulated *M*. *separata* herbivory-induced defense response in the *Coix* seedlings by analyzing the anti-herbivore traits such as RNA-seq data. Our results indicated that the *M*. *separata* herbivory could induce defense response in the *Coix* leaves.

The meta-analysis transcriptomic response offers the possibility to reveal the response of *Coix* to the simulated *M*. *separata* herbivory. Simulated *M*. *separata* feeding led to largely distinct transcriptomic changes in local leaves, as shown in Fig 2, much more numbers of DEGs were induced in NP1 group than in the NP6 group. In the common DEGs of NP1 group and NP6 group, genes were related to wounding and regulation. We further analyzed the function of these DEGs, most of which were related to defense and stress response, suggesting that *Coix* herbivore induced larger transcriptome changes.

**Fig 3:**
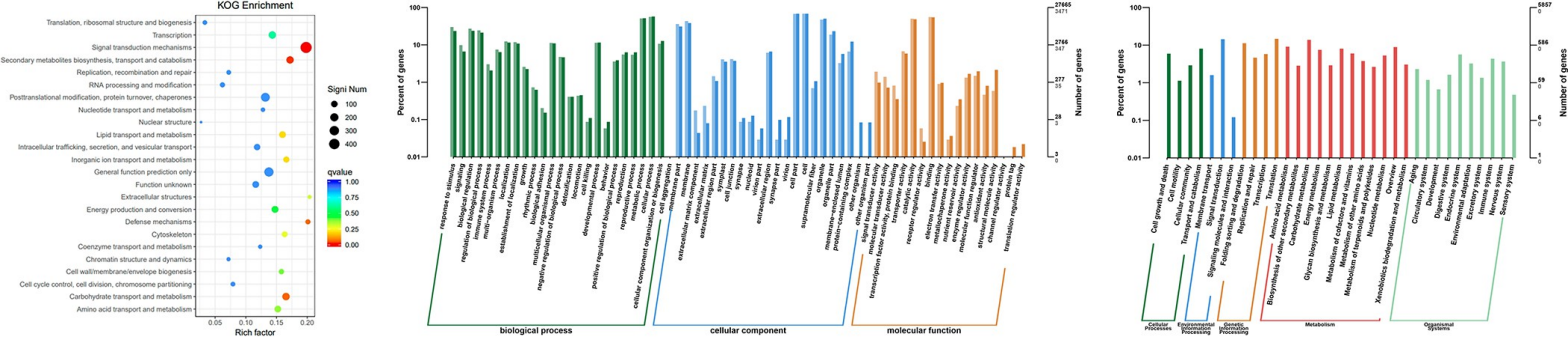
The functional analysis of the herbivory responses of the *Coix* leaves. (A) The KOG functional classification of all DEGs after W+OS treatment 1h in *Coix lacryma-jobi* L.Left: The KOG functional classification. Right: The number of DEGs mapped into each KOG functional classification. (B) Analysis of Gene Ontology (GO) function enrichment of the DEGs after W+OS treatment 1h, which represented the herbivory response DEGs. The functions of genes covering three main categories, including biological processes (BP), cellular components (CC) and molecular functions (MF). X-ray: Functional classification; Y-ray (right): The number of annotated genes (the numbers above) and the number of DEGs (the numbers below); Y-ray (left): The percent of genes. Different colored columns reprensent different functional classifications. The light colored columns represent DEGs, the deep colored columns represent all the annotated genes. (C) Description of the mapped KEGG pathways of the annotated genes after W+OS treatment 1h in *Coix lacryma-jobi* L. Left: The KEGG pathways; Right: The number of genes mapped to each KEGG pathway.

To investigate the functions of the herbivory responses of the *Coix* leaves, gene ontology (GO) and EuKaryotic Orthologous Groups (KOG) analyses were performed to describe the function of the DEGs. The categories “the general functional prediction only”, “signal transduction mechanisms”, “posttranslational modification, protein turnover, chaperones”, “carbohydrate transport and metabolism” and “secondary metabolites biosynthesis, transport and catabolism” were enriched in KOG analysis (Fig 3A and S3 Table). In the GO terms, the enriched biological processes from the herbivory response regulated genes in the fourth leaves of the NP1 group included ‘response to stimulus’, ‘response to stress’, ‘cellular response to stimulus’, ‘defense response’ and ‘response to chemical’ (S4 Table and Fig 3B). Genes in GO and KOG functional groups were strikingly more abundant, which provides basic information for further analysis of herbivory stress mechanisms in *Coix lacryma-jobi* L. Meanwhile, we performed enrichment analyses by mapping the sequences to the Kyoto Encyclopedia of Genes and Genomes (KEGG) database categories. The genes with KEGG annotation were assigned to 33 classes with a threshold of P-value<0.05 (S5 Table), mainly related to transport and catabolism, signal transduction, folding sorting and degradation, translation and carbohydrate metabolism. KEGG enrichment analyses also indicated that the annotated genes were significantly enriched in the main pathways of environmental information processing (ko04075), genetic information processing (ko03013) and metabolism (ko01110), which were closely related to stress (Fig 3C). Thus, wounding and OS (W+OS) treatment induced much starker transcriptome changes after 1h than 6h in *Coix* leaves.

### The regulation of transcription factors responding to the simulated herbivory

**Fig 4:**
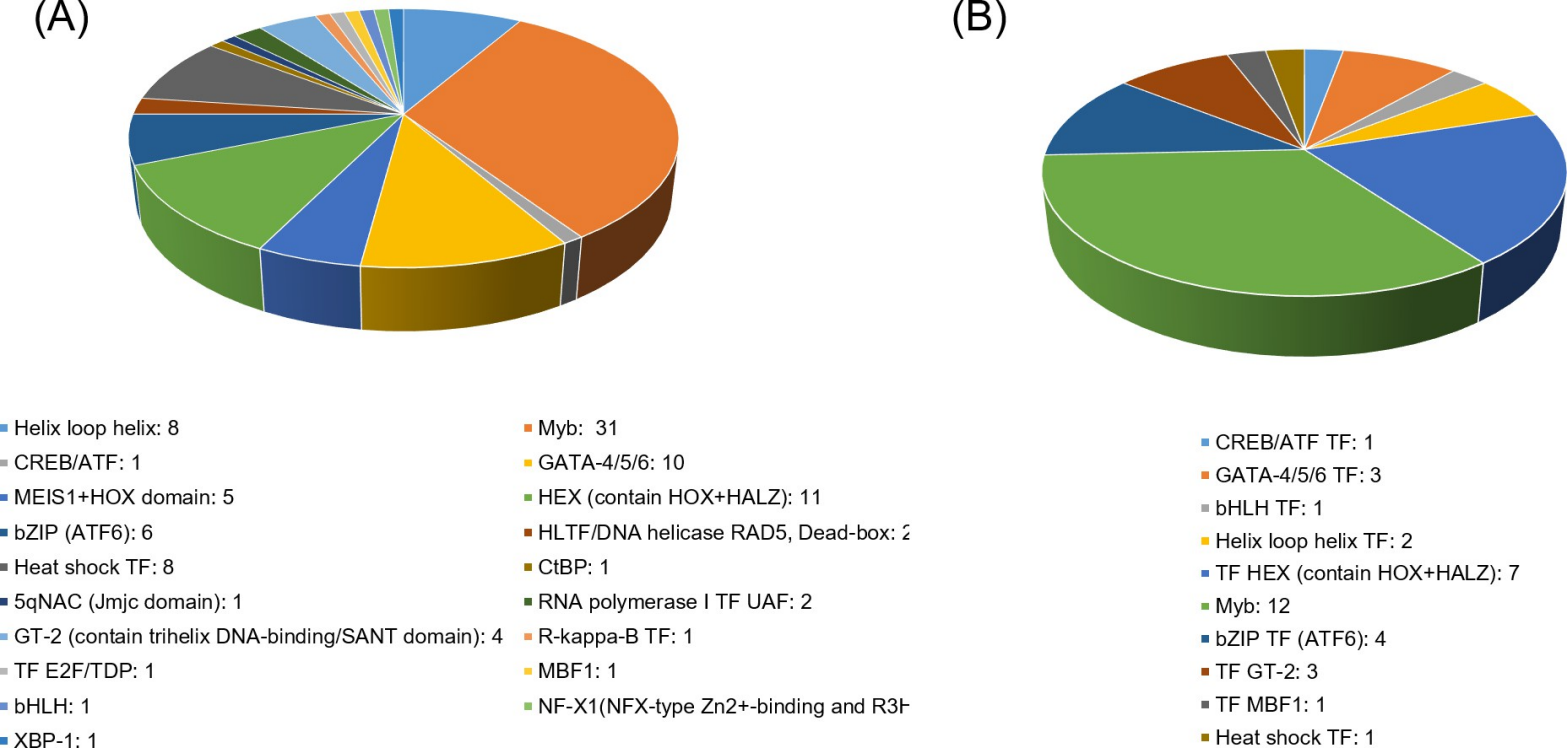
The differentially expressed TF families in *Coix lacryma-jobi* L. after W+OS treatment 1h (A) and 6h (B).

The differentially regulated transcription factors (TF) were performed by searching the Plant Transcription Factor Database (http://planttfdb.gao-lab.org/). We identified 96 and 35 unique TFs from 19 different families, which were from the samples treated after 1h and 6h, respectively (S6 Table). The most abundant TF families were Myb (31 DEGs), GATA-4/5/6 (10 DEGs), Helix loop helix (8 DEGs), Heat shock protein (8 DEGs) and bZIP family (6 DEGs) in the samples of 1h post-treatment (Fig 4A). Among the 96 differentially regulated transcription factors from the samples treated after 1h, 58 TFs were upregulated and 38 TFs were downregulated (S6 Table). Six hours after treatment, the most abundant TF families were Myb (12 DEGs), HEX (7 DEGs), bZIP (4 DEGs), GT-2 (3 DEGs) and GATA-4/5/6 (3 DEGs), which exhibited less number of TFs than those of 1h treatment (Fig 4B). Among the 19 TF families, 10 families were commonly regulated, including helix-loop-helix, Myb, CREB/ARF, GATA-4/5/6, bZIP, Heat shock protein, GT-2, MBF1, bHLH, HEX at 1h and 6h after wounding and OS treatment (W+OS). Among the 35 differentially regulated transcription factors from the samples treated after 6h, 28 TFs were upregulated and 7 TFs were downregulated (S6 Table). It is possible that these TFs regulated the transcriptional responses to insect herbivory in the early (1h) and later (6h) time points.

### *M*. *separata* herbivory can prime *Coix* systemic leaves for increased resistance to *M*. *separata*

**Fig 5:**
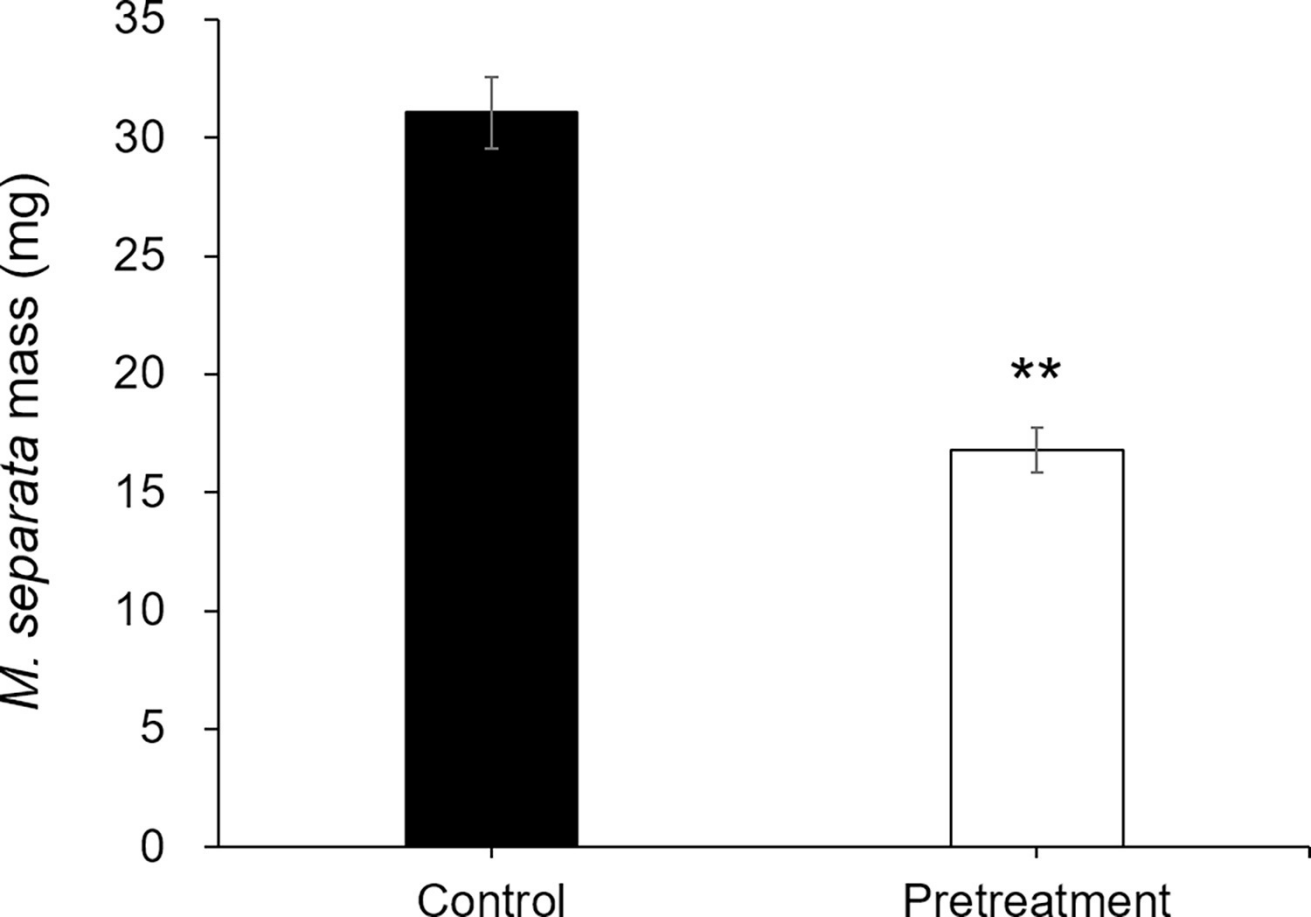
*M*. *separata* masses (mean ±s.e.; n = 20) after feeding for 3d on the systemic leaves of OS pretreated and control plants. Asterisks represent significant differences between pretreated and control samples (Student’s t-test; *, p<0.05, **, p<0.01).

To further investigate the defense responses of the systemic leaves to *M*. *separata*, *Coix* plants were treated with W+OS on leaf 3 once a day for three consecutive days, and after 4d, *M*. *separata* larvae were allowed to feed on leaf 4 for 3d before their masses were recorded. Compared with the control group, caterpillars fed on the pretreated plants gained 46% less average mass (Fig 5), suggesting the systemic defense response of the plants to the subsequently infested caterpillars.

**Fig 6:**
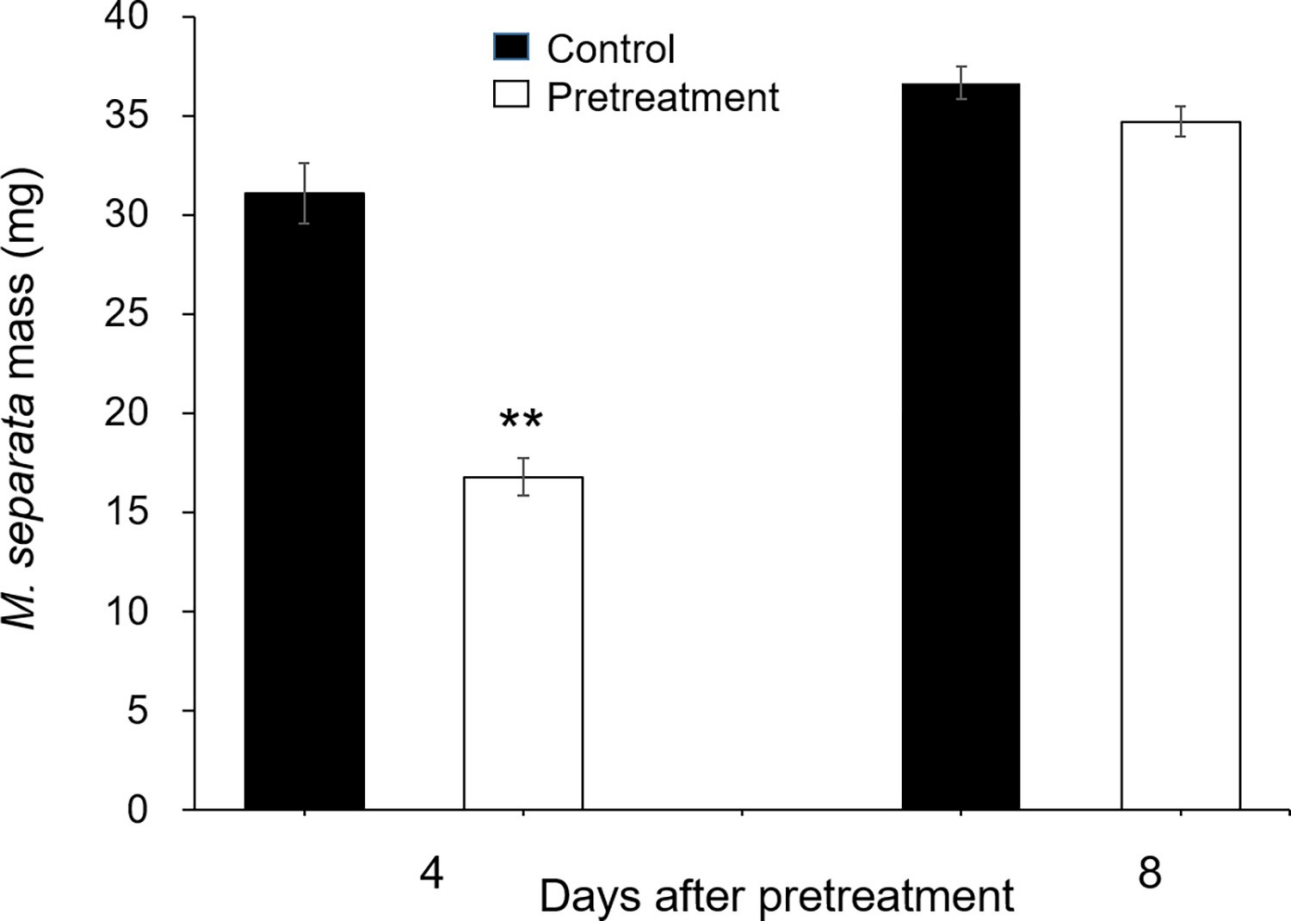
Simulated herbivory primes *Coix* systemic leaves for enhanced resistance. The third leaves of *Coix* seedlings were pretreated with W+OS for four days (pretreatment group), while in the control group, the leaves were untreated. The plants were rested for another 4, or 8 d before *M*. *separata* larvae were infested on the fourth leaves (one neonates/plant). The masses of insects after 3d of feeding were recorded. Data are mean ±SE; Student’s t-test; **P<0.05; n = 20.

Many studies have indicated herbivory can activate priming in systemic leaves. We sought to determine whether *M*. *separata* herbivory on *Coix* primes systemic leaves for enhanced resistance. The third leaf of each *Coix* seeding was pretreated with 20μL of *M*. *separata* OS. Four and eight days (resting time) after the larvae treatment, *M*. *separata* larvae (1d old and reared on maize seedlings since hatching) were infested on the fourth leaves of these pretreated *Coix* plants. The control *Coix* plants, which were not pretreated, were similarly infested with *M*. *separata* larvae. The insects were allowed to feed for 3d before their masses were recorded. Caterpillars fed on the pretreated plants, which had 4 d of resting, gained 54% of the masses of those fed on the control plants, while caterpillars fed on the pretreated plants, which had 8 d of resting, had no great difference from those fed on the control plants (Figs 5 and 6).

### *M*. *separata* herbivory defense priming related to Jasmonic acid

**Fig 7:**
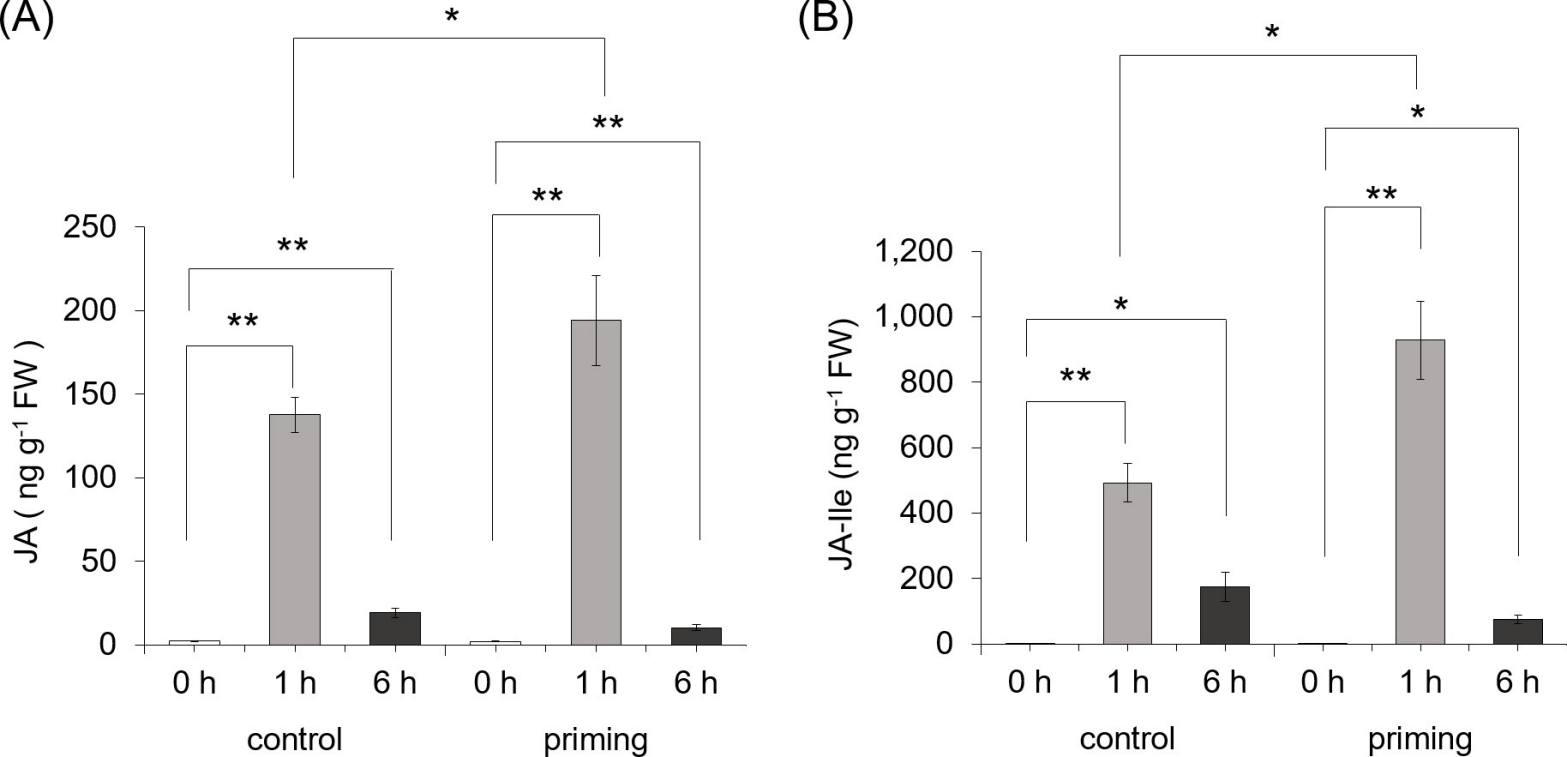
*M*. *separata* herbivory primes systemic leaves for increased JA and JA-Ile. In the priming group, *Coix* third leaves were infested with *M*. *separata* larvae (one/plant) for four days and then remove the caterpillars. After resting for 4d, the fourth leaves were treated with W+OS, samples of fourth leaves were collected at 0h, 1h and 6h for analyzing JA and JA-Ile contents. While in the control group, the third leaves were untreated, the fourth leaves were treated with W+OS and were collected at 0h, 1h and 6h for analyzing JA and JA-Ile contents. (means ±S.E., n = 6).

As a phytohormones, JA plays important roles in regulating defensive responses. Therefore, we speculated that simulated herbivory on third leaves could prime the fourth leaves for enhanced JA response to subsequent OS treatment. *Coix* seedlings (third leaves) were first pretreated with OS, four days after pretreatment, the systemic leaves of the plants from the pretreated group and control group were treated with OS and samples were harvested after 0 h, 1 h and 6 h. It was found that the levels of JA in the systemic leaves, which were harvested after 1 h, were 41% higher in the priming group than in the control group, while the levels of JA in the systemic leaves, which were harvested after 6 h, were almost the same in the priming group and the control group (Fig 7A). Similar results were found for the actual functional jasmonate, JA-Ile. The levels of JA-Ile in the systemic leaves, which were harvested after 1 h, were 89% higher in the priming group than in the control group, while the levels of JA-Ile in the systemic leaves, which were harvested after 6 h, were almost the same in the priming group and the control group (Fig 7B). Thus the levels of JA and JA-Ile in the systemic leaves, which were harvested after 1 h, were significantly different in the priming group and the control group, while were not in the systemic leaves, which were harvested after 6 h.

The present study is the first measurement of JA in *Coix* systemic leaves. Traditionally, the JA has been associated with plant responses to chewing herbivores and herbivore defense priming is JA-dependented. Our results demonstrate that the JA and JA-Ile were elicited in systemic leaves of *Coix* after simulated *M*. *separata* feeding treatment, which is consistent with many other studies reported in maize [7], wild tobacco [31] and Arabidopsis [42]. Meanwhile, we detected that the level of JA was higher at 1h than 6h after W+OS treatment. Many measurements from dicotyledonous plants have revealed the systemic signals travels faster than monocotyledonous plant. For example, the studies of *N*.*attenuata* [43] and Arabidopsis [42] indicated that the systemic accumulation of JA or JA-Ile within 15min, the speed of the mobile signal translocation was in the range 3.4–4.5cm min^-1^[44]. The measurements in maize revealed that the systemic jasmonate synthesis was slower than in *N*. *attenuata* or Arabidopsis, 15-30min was needed for it to exit the local leaf [7]. Our results also indicated simulated *M*. *separata* feeding induced much higher level of JA and JA-Ile at 1h and then decreased at 6h, which is consistent with the previous study in Arabidopsis, in which genes in the JA signaling pathway accumulated within 15 min after wounding, attained peak levels 30–60 min and then declined, while the JA-Ile levels in damaged leaves remained high for 3–6 h after wounding [42].

Although the pretreatment of the third leaves strongly primed *Coix* for enhanced levels of JA (Fig 7A), only a few of JA biosynthetic genes fitted into the transcriptional pattern of priming. We inspected the expression levels of JA biosynthetic genes in P1-C and NP1-C groups (S1 Fig). In the previous studies, among JA biosynthetic genes, JAR1 protein are differentially induced upon wounding or pathogen challenge [45]. *Opr7* and *opr8* double mutants have reduced levels of JA accumulation and required for resistance to herbivorous insects in maize [46]. Moreover, JA synthesis/signaling genes such as *LOX3*, *LOX4*, *LOX5*, *LOX6*, *LOX7/8*, *LOX9*, *LOX10*, *AOS1*, *AOC*, *OPR1*, *OPR2* were attenuated in *opr7 opr8* double mutants in maize. In our result, only *LOX3* and *OPR5* genes were slightly increased in the fourth leaves of the P1-C group compared with the NP1-C group, while the rest of the JA biosynthetic genes seemed not to be involved in priming. Thus, post-transcriptional regulation of JA biosynthetic genes may involve in priming. The mechanism of *Coix* defense priming needs further study.

Previous studies have documented W+OS herbivory feeding duration but not the extent of damaged area plays an important role in activating priming in the systemic leaves [47]. We demonstrated that *M*. *separata* herbivory on *Coix* strongly primed systemic leaves for enhanced resistance and levels of JA. Similar results were also indicated in maize [47], the primed plants had higher concentration of JA than non-primed plants. So, it is possible that the priming defense response was JA-regulated. The finding in Arabidopsis repetitive touching leaves also showed that the plants have increased resistance to fungus [48].

### Priming enhances transcriptional regulation of various defense-related genes in systemic leaves in response to W+OS treatment

To investigate the underlying molecular mechanism of defense priming, we performed a global gene expression analysis on the *Coix* seedlings with RNA-seq. The W+OS pretreated and untreated on the third leaves were allowed to rest for 4d, then their fourth leaves were all treated with OS. After 1h (NP1 and P1, the third leaves were non-primed and the fourth leaves were W+OS treated and collected after 1h, named NP1, the third leaves were primed and the fourth leaves were W+OS treated and collected after 1h, named P1) and 6h (NP6 and P6, the third leaves were non-primed and the fourth leaves were W+OS treated and collected after 6h, named NP6, the third leaves were primed and the fourth leaves were W+OS treated and collected after 6h, named P6), the fourth leaves of the four groups were collected for RNA-seq. Plants without any pretreatment and OS treatment were used as control.

**Fig 8:**
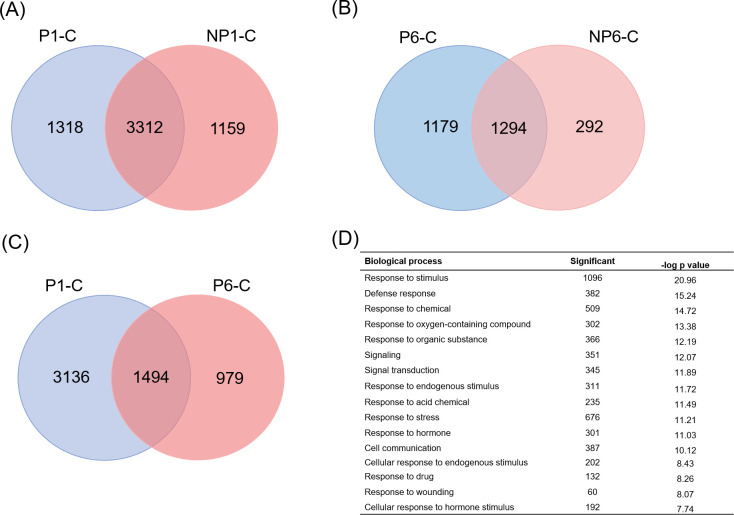
Primed *Coix* leaves exhibit transcriptional changes responding to the insect herbivory. (A, B, C) Venn diagram depicting the commonly and specifically regulated DEGs of P1-C (the third leaves were primed and the fourth leaves were W+OS treated and collected after 1h, named P1) & NP1-C (the third leaves were non-primed and the fourth leaves were W+OS treated and collected after 1h, named NP1) (A), P6-C (the third leaves were primed and the fourth leaves were W+OS treated and collected after 6h, named P6) & NP6-C (the third leaves were non-primed and the fourth leaves were W+OS treated and collected after 6h, named NP6) (B) and P1-C & P6-C (C). (D) Enriched GO terms (biological process) from the DEGs unique for the P1-C group.

Transcriptional analysis was performed in the two unpretreated groups (NP1and NP6) and two pretreated groups (P1 and P6) in this study. Totally, 13,160 DEGs were identified in the transcriptome sequencing (S7 Table). The fourth leaves of the NP1 group, which only treated with OS, had 4,471 DEGs (2262 up- and 2209 down-regulated), while in the primed P1 group, which were pretreated with OS, had 2,433 up- and 2,207 down-regulated genes in the fourth leaves. Meanwhile, the fourth leaves of the NP6 group, had 1586 DEGs and the primed P6 group had 2,473 DEGs (S7 Table). Venn diagrams indicated that the fourth leaves of the P1-C and NP1-C groups had 3,312 common DEGs, among which 1,428 DEGs (43.1%) exhibited at least 10% increased (1,204 genes) or decreased (224 genes) transcript levels in the P1-C group than in the NP1-C group, and 526 DEGs were found to have at least 50% increased (440 genes) or decreased (86 genes) transcript levels in the P1-C group than in the NP1-C group (S8 Table). Specially, in the P1-C group 1,318 DEGs were up- or down-regulated, while in the non-primed NP1-C group there were 1,159 specifically regulated DEGs (Fig 8A). Similarly, the P6-C and NP6-C were compared and the result were indicated in Fig 8B. The fourth leaves of the P6-C and NP6-C had 1,294 common DEGs, among which 999 DEGs (77.2%) exhibited at least 10% increased (449 genes) or decreased (550 genes) transcript levels in the P6-C group than in the NP6-C group (S9 Table). Specially, in the P6-C group, 1,179 genes were specifically regulated and in the non-primed NP6-C there were only 292 genes. Meanwhile, the DEGs of P1-C and P6-C groups were compared and the venn diagrams were shown in Fig 8C. These data suggested that pretreatment on the third leaves could largely elicit the transcriptional changes of the fourth leaves.

The present study was the first analysis the effect of priming on *Coix* transcriptome changes in the leaves. The primed P1 and P6 groups exhibited many induced genes compared with the NP1 and NP6 groups (Fig 8A and 8B). The transcriptome data showed that 28.5% of the total DEGs in the P1 group and 47.7% in the P6 group were specifically regulated by priming. Among the common DEGs of P1-C and NP1-C, 43.1% (1,428 genes) of DEGs in the P1-C group showed further up- or down-regulated (at least 10%) if the third leaves were pretreated. Thus, we hypothesize that in addition to the 1,318 specifically regulated genes in the fourth leaves of the P1-C group, the 3,312 genes maybe contributed to induce/suppress priming in the priming-induced defense responses. However, the mechanism of how the systemic fourth leaves were primed against *M*. *separata*, such as transcriptome alternation and enhanced JA accumulation, remains unclear. Previous studies indicated that epigenetic changes had been detected in stress-treated plants, such as pathogen infection. The biotic stress led to methylome reconfigurations of genome, which were associated with transcriptome changes [49]. In our *Coix*-*M*. *separata* interaction system, it is likely that the *Coix* epigenetic changes after herbivory involved.

Then, in order to examine the function of the priming-related DEGs, GO analysis was performed. The enriched biological processes from the common DEGs in the fourth leaves of the P1-C and NP1-C groups included ‘response to stimulus’, ‘response to stress’, ‘cellular response to stimulus’, ‘response to chemical’, and ‘cell communication’ (S10 Table). While GO terms enriched from the unique DEGs regulated in the fourth leaves of the P1-NP1 group included ‘small molecule metabolic process’, ‘oxoacid metabolic process’, ‘organic acid metabolic process’, ‘carboxylic acid metabolic process’ and ‘cellular lipid metabolic process’ (S11 Table). The uniquely regulated genes in the fourth leaves of the P1-C group included ‘response to stimulus’, ‘defense response’, ‘response to chemical’, ‘oxygen-containing compound’ and ‘response to organic substance’ (Fig 8D).

Given that the accumulation of JA was regulated by priming, we inspected the expression levels of JA biosynthetic genes in P1-C and NP1-C groups. Strikingly, among JA biosynthetic genes, only LOX3 and OPR5 genes were slightly but significantly increased in the fourth leaves of the P1-C group compared with the NP1-C group, while the rest of the JA biosynthetic genes seemed not to be induced (S1 Fig).

Previously, it was found that many defense priming against herbivory was induced by HIPVs, oviposition, β-amino-butyric acid, or cytokinin [10,50,51]. Studies in various species have documented that priming is a general trait of the defense system. For example, HIPVs such as indole primed the neighboring plants for increased release of defense volatiles and expression of defense genes compared with the untreated maize and rice [52,53]. Similarly, simulated herbivory in maize had a strong effect on responses of systemic leaves and the pretreatment on third leaves primed the enhanced defense in the fourth leaves, including increased JA [7,47]. Meanwhile, it was also reported the herbivory-induced priming signals conveyed by the vasculature, for example, the belowground herbivory of *D*. *v*. *virgifera* induced the resistance to *S*. *littoralis* in maize leaves, and these leaves were primed for elevated chlorogenic acid in the subsequent *S*. *littoralis* feeding [54].

It has been documented that consecutive and repetitive wounding or herbivory were necessary for successfully inducing defense priming. For example, repeated *M*. *separata* OS treatment to one row of wounds once a day on two consecutive days in third maize leaves primed the systemic fourth leaves, in contrast, applying OS to one or even four rows of wounds in third leaves only once did not find any priming effect in the systemic leaves [47]. This was consistent with the findings in Arabidopsis and wild tobacco [55,56]. In this study, we applied 20 μL of *M*. *separata* OS at a row of puncture wounds and repeated once a day for 3 days to ensure the priming induced. Although pretreatment strongly primed plants for enhanced defense response than non-primed ones in the presence of stresses, it is unclear whether only wounding without OS would prime the systemic defense and is still unknown how repetitive wounding or herbivory primes the systemic defense.

The systemic priming was dependent on perception of elicitors in the *M*. *separata* OS [47]. Previous studies have documented that fatty acid–amino acid conjugates (FACs) were known to be potent elicitors in various insect OS [57], while *M*. *separata* OS are rich in several types of FACs [28]. Therefore, it is important for *Coix* to percept of FACs in the *M*. *separata* OS so as to induce the priming agents. Although it is still unclear which was the mobile priming agents and how they promoted the plants to a primed state, many studies shown that these agents were likely herbivory-induced systemic signals, such as Ca^2+^, reactive oxygen species, and ion channels [58,59].

### Validation of RNA-seq results by qPCR

**Fig 9:**
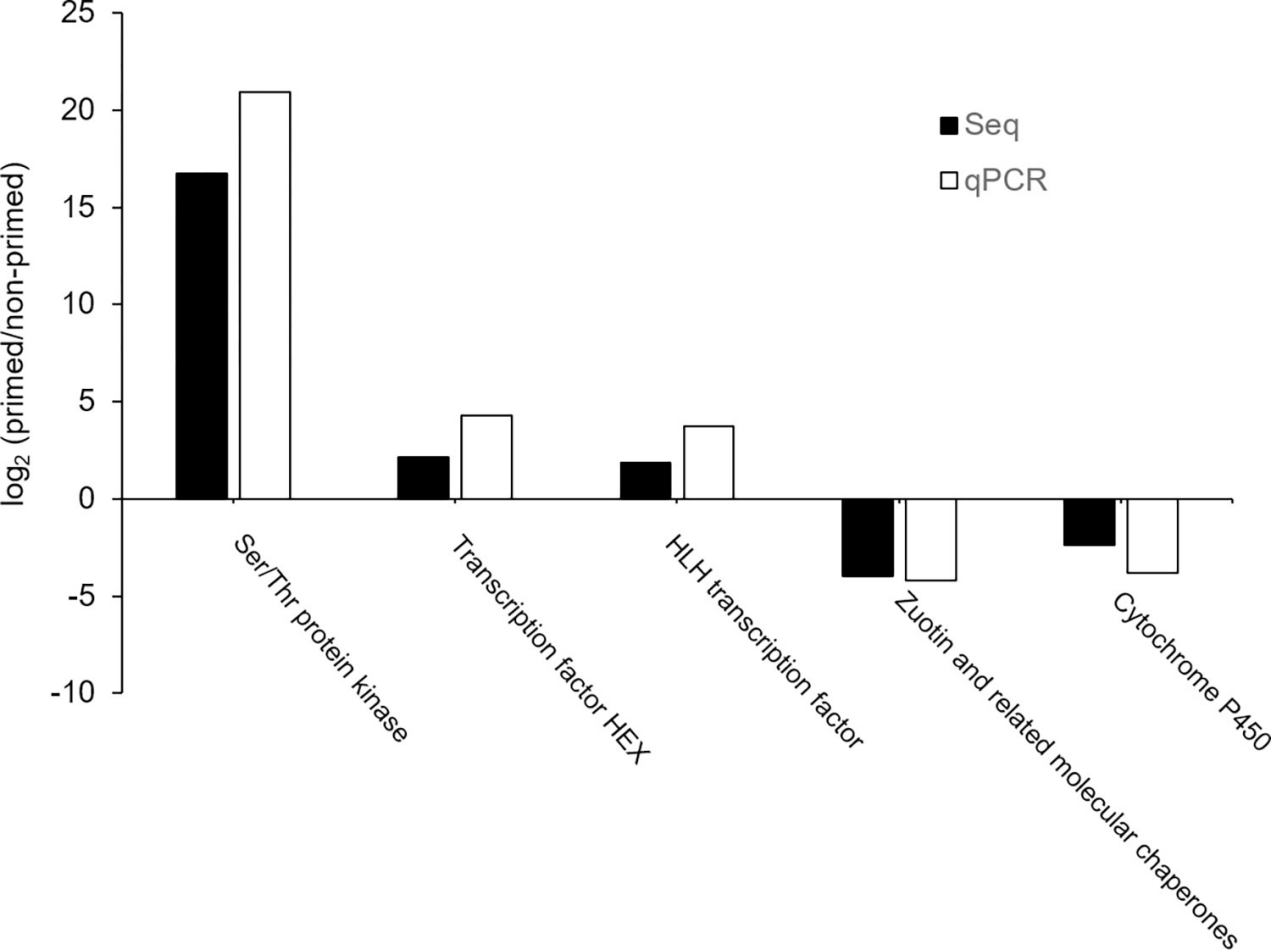
Expression levels of defense-related genes in qRT-PCR and RNA-seq.

The RT-qPCR was performed with selected five herbivory responded genes, including helix loop helix transcription factor, protein cytochrome P450, transcription factor HEX, Zuotin and related molecular chaperones and serine/threoine protein kinase, to validate the RNA-Seq results. The expression patterns of the qPCR were consistent with the bioinformatics data (Fig 9), which validated the results of the RNA-Seq.

## Conclusions

In this study we first showed the simulated *M*. *separata* herbivory-induced defense responses in *Coix* systemic leaves and *Coix* is able to sense the repetitively simulated herbivory and induce priming in the systemic leaves, so as to enable the systemic leaves to respond to the successive *M*. *separata* herbivory with highly increased JA levels. Also, the priming boosts the transcriptional changes in the systemic leaves. These results provide new insight into *Coix* herbivory and priming, which is important for *Coix* defense against *M*. *separata*. However, the defensive metabolites involved in the defensive response in the *Coix* still had not been studied, and the mechanism by which JA regulates systemic leaves to confer resistance need further investigation. Further studies uncovering the mechanism of priming could promote breeding of *Coix* with enhanced resistance to insects.

## Supporting information

S1 Fig JA biosynthesis and catabolism genes in control, NP1, NP6, P1 and P6 groups do not have different transcript levels. *Coix* third leaves were untreated or pretreated with W+OS for 3 consecutive days. After 4 days of resting, the fourth leaves were treated with W+OS. After another 1h and 6 h, samples of fourth leaves were collected for analyzing global transcriptomic changes. The relative transcript levels of genes involved in JA biosynthesis and catabolism were retrieved from the RNA-seq data (n = 3).
(TIF)

S1 Table *Coix* herbivory candidate genes primers. (DOCX)

S2 Table All DEGs in *Coix* after simulated *M*. *separata* herbivory 1h and 6h. (XLSX)

S3 Table KOG functional classification of DEGs after W+OS treatment 1h. (XLSX)

S4 Table Enriched GO terms of DEGs in NP1-C. (XLSX)

S5 Table KEGG pathway of the DEGs in NP1-C. (XLSX)

S6 Table Differentially regulated transcription factors in NP1 and NP6. (XLSX)

S7 Table All the DEGs of primed and non-priming groups. (XLSX)

S8 Table The comparison of the Mean TPM of common DEGs in P1-C and NP1-C groups. (XLSX)

S9 Table The comparison of the Mean TPM of common DEGs in P6 and NP6 groups. (XLSX)

S10 Table The enriched biological processes from the common DEGs of P1-C & NP1-C. (XLSX)

S11 Table The enriched biological processes from the unique DEGs of P1- NP1. (XLSX)
