# Supplementary figures and images for: Mythimna separata herbivory primes Coix resistance in systemic leaves
Source: PLoS One. 2024 Nov 4;19(11):e0313015. doi: 10.1371/journal.pone.0313015 (PMC11534230; doi:10.1371/journal.pone.0313015)

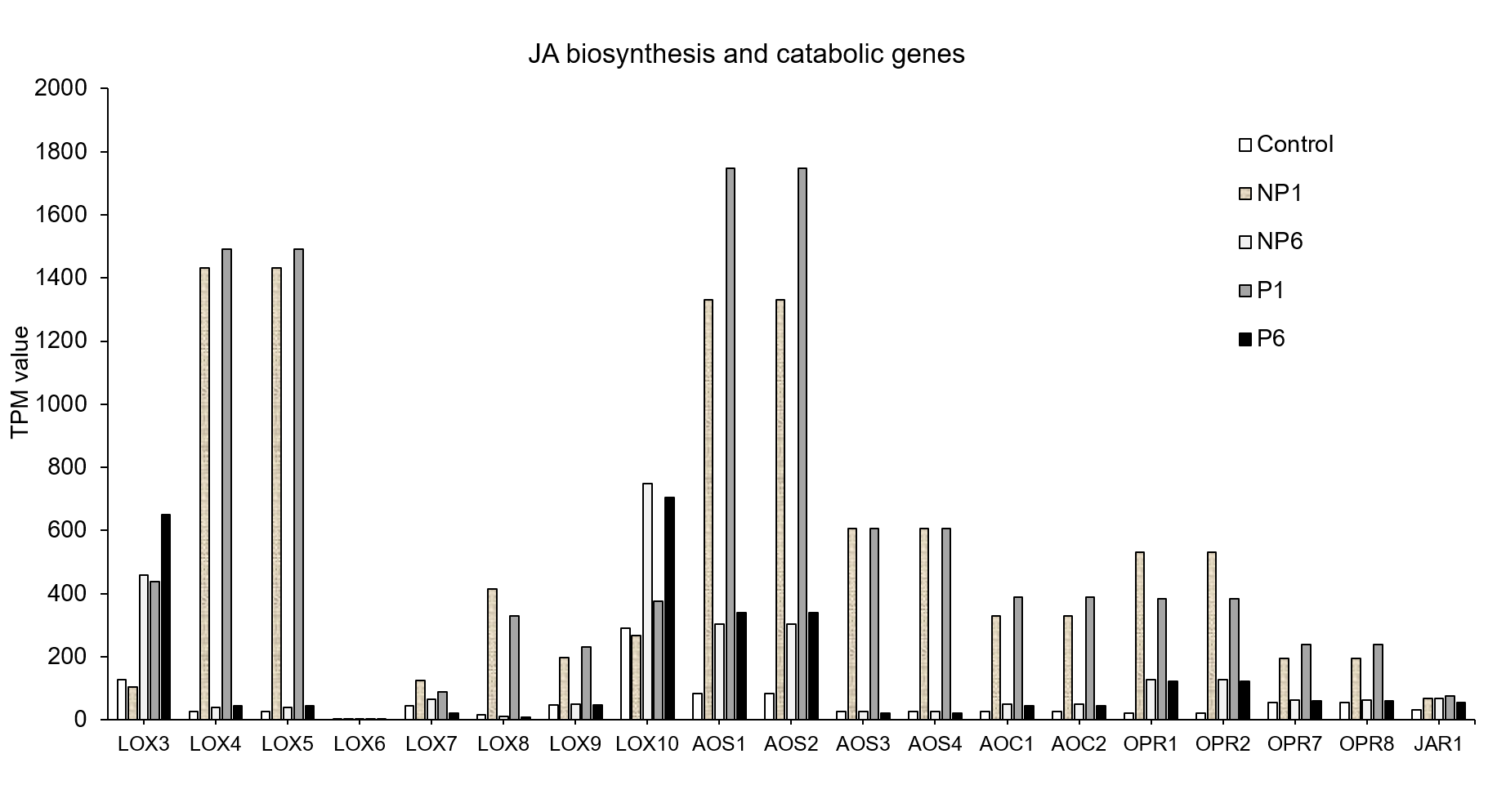

Supplement: S1 Fig — Coix third leaves were untreated or pretreated with W+OS for 3 consecutive days. After 4 days of resting, the fourth leaves were treated with W+OS. After another 1h and 6 h, samples of fourth leaves were collected for analyzing global transcriptomic changes. The relative transcript levels of genes involved in JA biosynthesis and catabolism were retrieved from the RNA-seq data (n = 3). (TIF) [file pone.0313015.s001.tif]
