# Supplementary material for: Mythimna separata herbivory primes Coix resistance in systemic leaves
Source: PLoS One. 2024 Nov 4;19(11):e0313015. doi: 10.1371/journal.pone.0313015 (PMC11534230; doi:10.1371/journal.pone.0313015)
Supplement: S1 Table — (DOCX) [file pone.0313015.s002.docx]

**S1 Table. *Coix* herbivory candidate genes sequence-specific primers used for qRT-PCR**

| **Gene ID** | **Annotation** | **Primer-F (5'-3')** | **Primer-R (5'-3')** |
| --- | --- | --- | --- |
| Cl014393 | *Cytochrome P450* | TCACGGAGGCAGAGTTAGT | CTGGACGACATGGCTCGA |
| Cl005682 | *Helix loop helix transcription factor* | TGGATGACTTCTGGGATGG | TTAGTTAGGTGACTTGCGAC |
| Cl035816 | *Ser/Thr protein kinase* | GGCAAGCACCATTTCTACG | TCCTGTCCTGGCACATCC |
| Cl014089 | *Zuotin and related molecular chaperones* | ATGCCGACCGTCAGTATGG | TCTTCGGTTAGCCGCTC |
| Cl023335 | *Transcription factor HEX* | TTCAAGCCGCAGTTCGTGT | CGTTTCGTGGAGGATGCTGTC |
